# Exploring the relationship between smoking and poor sleep quality: a cross-sectional study using NHANES

**DOI:** 10.3389/fpsyt.2024.1407741

**Published:** 2024-05-28

**Authors:** Haoxiong Sun, Sijia Li

**Affiliations:** ^1^ Independent Researcher, Melbourne, VIC, Australia; ^2^ Drug Discovery Biology, Monash Institute of Pharmaceutical Sciences, Melbourne, VIC, Australia

**Keywords:** sleep disorder, smoking, nicotine, snoring, sleep insufficiency

## Abstract

**Introduction:**

Sleeping disorders is a high prevalent disorder, and although previous research has suggested a link between smoking and sleep disorders, there is a lack of large-scale, nationally representative studies examining this association across multiple sleep outcomes and exploring dose-response relationships.

**Methods:**

This study used data from 30,269 participants from the NHANES database (2007–2020). Weighted logistic regression models were used to assess the associations between smoking status (non-smoker, light smoker, moderate smoker, and heavy smoker) and various sleep outcomes, including insufficient sleep duration, reported sleep problems, snoring, snorting, or stopping breathing during sleep, and daytime sleepiness. Dose-response relationships were explored using restricted cubic splines.

**Results:**

Compared to non-smokers, heavy smokers had significantly higher odds of experiencing insufficient sleep duration with OR 1.732 (95% CI 1.528–1.963, P <0.001), reported sleep problems with OR 1.990 (95% CI 1.766–2.243, P <0.001), occasional or frequent snoring with OR 1.908 (95% CI 1.164–3.128, P = 0.03), and occasional or frequent snorting or stopping breathing during sleep with OR 1.863 (95% CI 1.183–2.936, P = 0.022), while results for sometimes, often or almost always being overly sleepy during the day with OR 1.257 (95% CI 0.872–1.810, P = 0.115) are not significant. A trend of positive correlation was observed between smoking and all sleep disorder outcomes (P for trend < 0.05). Dose-response analyses revealed that the odds of these sleep outcomes increased with higher smoking levels.

**Conclusion:**

Smoking is significantly associated with various sleep disorders, and a dose-response relationship exists between smoking levels and the odds of experiencing these sleep problems. These findings underscore the importance of addressing smoking as a modifiable risk factor for poor sleep health and suggest that reducing smoking, even if complete cessation is not achieved, may have positive effects on sleep outcomes.

## 1 Introduction

Sleep is an important physiological process that plays a crucial role in maintaining physical and mental well-being (1, 2). Poor sleep quality has been associated with various adverse health outcomes, including cardiovascular diseases (3), metabolic disorders such as diabetes (4), and cognitive impairments (5). Sleep disorders are prevalent in the general population, with an estimated 83.6 million adults in the United States experiencing some form of sleep or wakefulness disorder (6). In recent years, there has been growing interest in identifying modifiable risk factors for poor sleep quality (7).

Smoking is a global health issue associated with numerous chronic diseases and premature deaths (8). Although the diverse harmful effects of smoking on respiratory and cardiovascular health are well recognized (9), the relationship between smoking and sleep quality remains understudied. Although some studies have identified pathological and epidemiological links between smoking and sleep disorders, like previous research has suggested that smoking may disrupt sleep architecture and lead to sleep disturbances (10–12), there is a lack of large-scale, nationally representative studies examining this association across multiple sleep outcomes and exploring dose-response relationships. Understanding the relationship between smoking and sleep quality is crucial for developing targeted interventions and public health strategies to improve sleep health and overall well-being.

The National Health and Nutrition Examination Survey (NHANES) is a nationally representative survey conducted in the United States that collects comprehensive data on various health-related factors, including smoking habits and sleep parameters (13). By leveraging NHANES data from 2007–2020 March, we can explore potential associations between smoking and multiple sleep outcomes (14). In this study, we aim to investigate the association between smoking and poor sleep quality or sleeping disorders by using data from multiple NHANES survey cycles. We will also explore dose-response relationships between smoking and sleep outcomes while adjusting for important covariates. We hypothesize that smokers have a higher prevalence of poor sleep quality and various sleep disorders compared to non-smokers.

## 2 Method

### 2.1 Study subjects

This cross-sectional study used data from the National Health and Nutrition Examination Survey (NHANES), a nationally representative survey conducted by the Centers for Disease Control and Prevention (CDC) in the United States. NHANES employs a complex, multistage probability sampling design to assess the health and nutritional status of the civilian, non-institutionalized U.S. population (15).

Data from six consecutive NHANES cycles (2007–2008, 2009–2010, 2011–2012, 2013–2014, 2015–2016, and 2017- 2020 March) were combined for this study, yielding a total sample size of 66,148 participants. Among these, 30,269 subjects had complete data on smoking status (including non-smokers) and were included in the analysis. Subjects who did not provide information on their smoking status (n = 35,879) were excluded.

**Figure 1:**
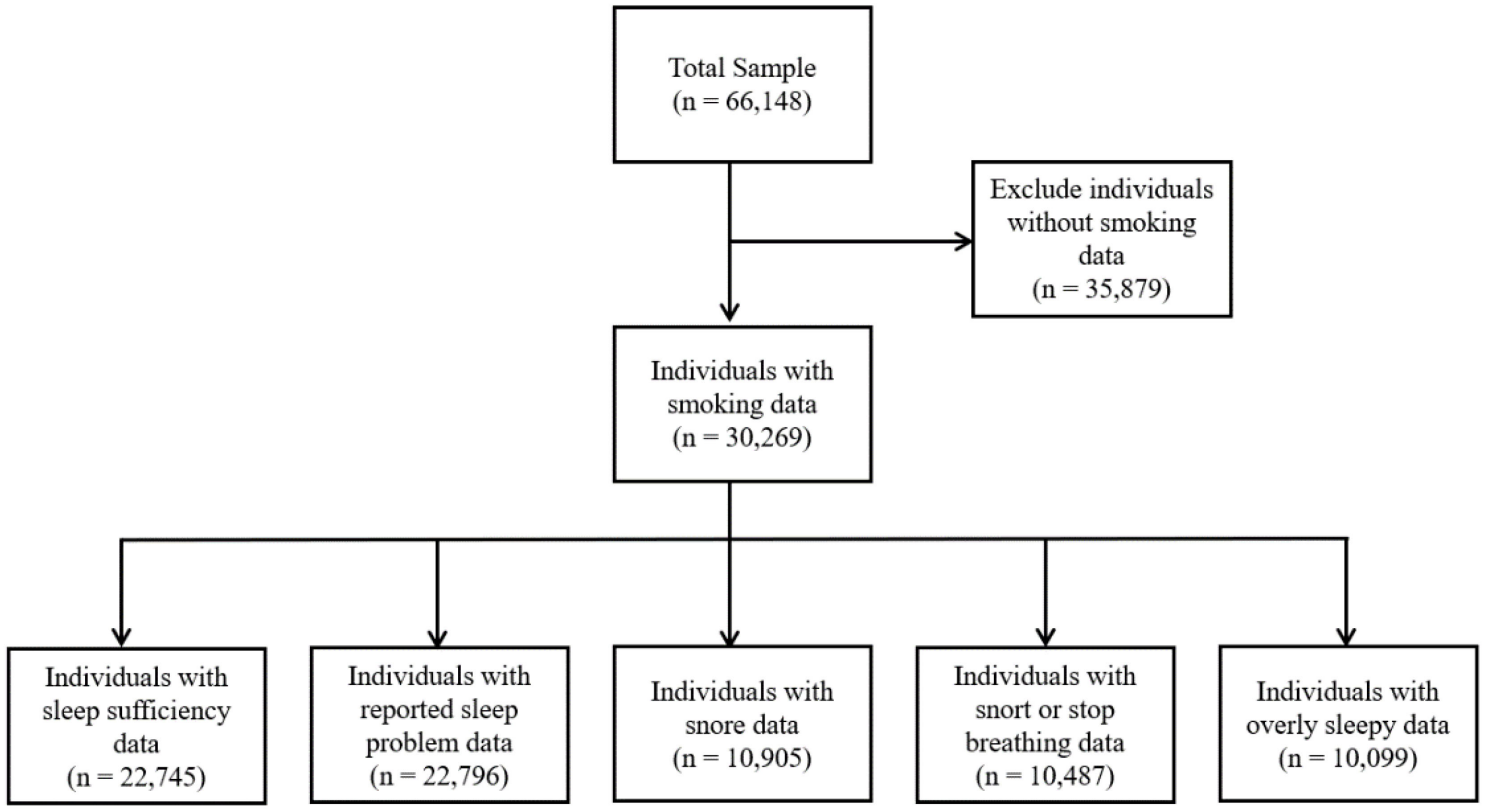
Flowchart of the sample selection from NHANES (2007–2020 March).

The study population varied depending on the sleep outcome of interest. For the analysis of sleep duration, 22,745 subjects with valid sleep duration data were included. The analysis of sleep problems included 22,796 subjects with available data. For the analyses of snoring, snorting or stop breathing during sleep, and overly sleepy, 10,905, 10,487, and 10,099 subjects were included, respectively, based on the availability of relevant data (**Figure 1**). Basic characteristics of included and excluded participants, including age, BMI, gender and race distribution were compared (**Supplementary Table S1**).

All participants provided written informed consent, and the NHANES study protocol was approved by the National Center for Health Statistics Research Ethics Review Board (15).

### 2.2 Exposure measurement

Smoking status was determined using the NHANES questionnaire item “SMQ020 - Smoked at least 100 cigarettes in life”. Participants who reported having smoked less than 100 cigarettes in their lifetime were classified as “non-smokers”. Among those who had smoked at least 100 cigarettes, we define the previous smoker by determining subjects whose answer for item “SMQ040 - Do you now smoke cigarettes?” is “3, Not at all”. For participants currently smoking, the intensity of smoking was assessed using the questionnaire item “SMD650 - Avg # cigarettes/day during past 30 days”.

Based on the responses to SMD650, smokers were further categorized into three groups: light smokers, who reported smoking more than 0 but fewer than 5 cigarettes per day, moderate smokers, who reported smoking more than 5 but fewer than 10 cigarettes per day and heavy smokers, who reported smoking 10 or more cigarettes per day.

For the dose-response analysis, the continuous variable derived from “SMD650 - Avg # cigarettes/day during past 30 days” was used to represent the average number of cigarettes smoked per day. This approach allowed for a more granular examination of the relationship between smoking quantity and sleep outcomes.

### 2.3 Outcome measurement

This study examined several sleep-related outcomes, including sleep duration sufficiency, diagnosed sleep problems, the frequency of snoring or snorting and stop breathing during sleep, and feeling overly sleepy during the day.

Sleep duration was assessed using two different questionnaire items across the NHANES cycles. In the 2007–2014 cycles, sleep duration was measured using the item “SLD010H - How much sleep do you get (hours)?”. For the 2015–2020 cycles, sleep duration was assessed using the item “SLD012 - Sleep hours”. Based on previous research, sleep duration was dichotomized into sufficient (>7 hours per night) and insufficient (≤7 hours per night) sleep (6, 14). Diagnosed sleep problems were determined using the questionnaire item “SLQ050 - Ever told doctor had trouble sleeping?”. Participants who reported being told by a doctor that they had trouble sleeping were considered to have a diagnosed sleep problem.

Snoring frequency was assessed using the questionnaire item “SLQ030 - How often do you snore?”. Responses were categorized as ‘never’, ‘rarely’ (1–2 nights per week), ‘occasionally’ (3–4 nights per week), and ‘frequently’ (5 or more nights per week).

The frequency of snorting or stop breathing during sleep was measured using the questionnaire item “SLQ040 - How often do you snort or stop breathing?”. Responses were categorized as ‘never’, ‘rarely’ (1–2 nights per week), ‘occasionally’ (3–4 nights per week), and ‘frequently’ (5 or more nights per week).

The frequency of feeling overly sleepy during the day was defined by “SLQ120 - How often feel overly sleepy during day?”, and Responses were categorized as ‘never’, ‘rarely’ (1 time a month), ‘sometimes’ (2–4 times a month), ‘frequently’ (5–15 times a month), and ‘almost always’ (more than 15 times a month).

In the dose-response analysis, we treated the ordinal categorical variables (snoring, snorting or stop breathing, and feeling overly sleepy) as binary variables. We defined ‘never’ and ‘rarely’ as 0, and ‘occasionally’ or more frequent categories as 1. The purpose of this approach was to investigate the relationship between smoking and the presence of occasional or more severe sleep problems, which we believe has more practical significance.

### 2.4 Covariable

Covariables were chosen based on previous research (13, 14). Demographic characteristics were extracted from the demographic questionnaire, including age, gender, race/ethnicity, marital status, family poverty income ratio, and education level. These variables were categorized as follows: Race/ethnicity: Non-Hispanic White, Non-Hispanic Black, Mexican American, Other Hispanic, and Other Race - Including Multi-Racial. Marital status: Married/living with partner, Widowed/divorced/separated, and Never married. Family poverty income ratio: Low income (<1), Middle income (1–3), and High income (≥3). Education level: Below high school, High school/GED, and College or above. Body mass index (BMI) was extracted from “BMXBMI - Body Mass Index (kg/m**2)”. BMI was treated as a continuous variable in the analysis. The exercise load is determined based on whether people have done 75–150 minutes of high-intensity exercise or 150–300 minutes of moderate-intensity exercise per week based on the physical activity guidelines for American (16). Subjects were categorized into lack of exercise (below the standard), adequate exercise (meeting the standard), or highly active exercise (exceeding the standard). Dietary energy intake was also considered. The recommended energy intake for males is 2000–3000 kcal per day, while for females, it is 1600–2400 kcal per day (17). Based on these recommendations, subjects were divided into three categories: low energy intake, adequate energy intake, or excessive energy intake. Medication status, including the presence of asthma, diabetes, heart failure, emphysema, and chronic bronchitis, was also considered as a covariate.

### 2.5 Statistical analysis

According to the NHANES protocol, all the data were integrated into a single dataset, and data analysis took into account the masked variance and applied the suggested weighting methodology. Sample weights from the Mobile Examination Center (MEC) interviews were used to address non-response, non-coverage, and unequal probabilities of selection (18).

Participants were divided into two groups based on sleep sufficiency or reported sleep problems, or divided into groups based on the frequency of snoring, snorting, or stopping breathing during sleep, or being overly sleepy during the day. To explore the differences between these groups, weighted descriptive analyses were performed. For continuous variables such as age and BMI, weighted percentages were computed. For categorical variables such as gender, race, education level, and smoking status, weighted percentages were computed, and the weighted chi-square test was used to assess group differences. Data are expressed as mean ± SE. Odds ratios (ORs) and 95% confidence intervals (CIs) for the association between sleep disorders and smoking status were calculated using weighted logistic regression models. Both unadjusted and multivariate adjusted models were used in this research: the crude model was adjusted for no covariates; Model 1 was adjusted for age, sex, and race/ethnicity; Model 2 was further adjusted for body mass index, education level, marital status, poverty level, exercise level, dietary energy intake level, and medication status.

To determine the association of smoking with sleep disorders, we performed the Cochran-Armitage trend test and used logistic regression models to test whether sleep disorder outcomes exhibited a positive trend across smoking levels, while adjusting for potential confounders.

The non-linear relationship between the amount of smoking and binary sleep outcomes was evaluated using restricted cubic splines. A dose-response curve was plotted to visualize the relationship, with the odds ratios and 95% CIs predicted for different smoking amounts using the Predict function.

All statistical analyses were performed using R software version 4.3.2 (http://www.R-project.org, The R Foundation). A p-value of < 0.05 was statistically significant.

## 3 Results

### 3.1 Baseline data for sleep disorders outcomes

#### 3.1.1 Baseline data for insufficient sleep duration

**Table 1** presents the weighted baseline characteristics of all participants, participants who have insufficient sleep duration, and participants who have sufficient sleep duration. Among the analyzed sample subjects, age, BMI, gender, race, education level, poverty level, marital status, exercise load, dietary energy intake, asthma, diabetes, emphysema, chronic bronchitis, and smoking status were associated with significant differences in sleep sufficiency.

**Table 1:** Baseline characteristics of participants by sleep duration sufficiency.

Variable	All participants	Insufficient Sleep	Sufficient Sleep	P-value
Age	48.753±0.278	46.833±0.169	46.195±0.139	0.004
BMI	28.732±0.040	29.436±0.071	28.356±0.048	<0.001
Gender				0.018
Male	48.753 (0.278)	50.339 (0.278)	51.731 (0.278)	
Female	51.247 (0.278)	49.661 (0.278)	48.269 (0.278)	
Race				<0.001
Mexican American	16.059 (0.204)	13.994 (0.193)	17.158 (0.210)	
Other Hispanic	10.836 (0.173)	11.366 (0.177)	10.554 (0.171)	
Non-Hispanic White	40.139 (0.273)	35.498 (0.266)	42.609 (0.275)	
Non-Hispanic Black	21.573 (0.229)	28.309 (0.251)	17.988 (0.214)	
Other Race	11.393(0.177)	10.832 (0.173)	11.692 (0.179)	
Education				<0.001
Below high school	25.957 (0.257)	26.227 (0.258)	25.806 (0.257)	
High school/GED	51.416 (0.293)	49.742 (0.293)	52.358 (0.293)	
College or above	22.626 (0.245)	24.031 (0.251)	21.836 (0.242)	
Poverty Level				<0.001
<1	23.809 (0.249)	24.662 (0.252)	23.352 (0.247)	
1-3	41.848 (0.289)	42.849 (0.289)	41.312 (0.288)	
>=3	34.343 (0.278)	32.490 (0.274)	35.336 (0.279)	
Marital status				<0.001
Married/living with partner	62.966 (0.271)	59.884 (0.275)	64.609 (0.269)	
Never married	14.245 (0.196)	15.177 (0.201)	13.748 (0.193)	
Widowed/divorced/separated	22.788 (0.236)	24.939 (0.243)	21.643 (0.231)	
Smoking				<0.001
Non-Smoker	56.379 (0.288)	52.865 (0.290)	58.330 (0.286)	
Former Smoker	23.319 (0.246)	22.449 (0.242)	23.803 (0.247)	
Light Smoker	5.097 (0.128)	5.636 (0.134)	4.798 (0.124)	
Moderate Smoker	3.681 (0.109)	4.258 (0.117)	3.361 (0.105)	
Heavy Smoker	11.523 (0.185)	14.793 (0.206)	9.707 (0.172)	
Exercise load				<0.001
Lack of Exercise	47.600 (0.324)	45.111 (0.323)	48.930 (0.324)	
Adequate Exercise	23.286 (0.274)	22.120 (0.269)	23.909 (0.277)	
Highly Active Exercise	29.114 (0.295)	32.769 (0.305)	27.161 (0.289)	
Diatery energy intake				<0.001
Low Energy Intake	42.164 (0.290)	42.951 (0.291)	41.744 (0.290)	
Adequate Energy Intake	36.865 (0.284)	34.347 (0.279)	38.207 (0.286)	
Excessive Energy Intake	20.971 (0.239)	22.702 (0.246)	20.049 (0.235)	
Asthma				<0.001
Yes	14.778 (0.197)	17.216 (0.210)	13.481 (0.190)	
No	85.221 (0.197)	82.783 (0.210)	86.519 (0.190)	
Diabetes				<0.001
Yes	11.868 (0.182)	13.190 (0.188)	11.166 (0.175)	
No	88.132 (0.182)	86.810 (0.188)	88.834 (0.175)	
Heart Failure				0.665
Yes	3.329 (0.105)	3.394 (0.101)	3.292 (0.099)	
No	96.671 (0.105)	96.606 (0.101)	96.708 (0.099)	
Emphysema				<0.001
Yes	2.128 (0.085)	2.512 (0.087)	1.912 (0.076)	
No	97.872 (0.085)	97.488 (0.087)	98.088 (0.076)	
Chronic Bronchitis				<0.001
Yes	5.504 (0.134)	7.053 (0.142)	4.632 (0.117)	
No	94.496 (0.134)	92.947 (0.142)	95.368 (0.117)	

#### 3.1.2 Baseline data for reported sleep problems

**Table 2** presents the weighted baseline characteristics of all participants, participants who reported sleep problems, and participants who never reported sleep problems. Among the analyzed sample subjects, age, BMI, gender, race, education level, poverty level, marital status, exercise load, dietary energy intake, asthma, diabetes, heart failure, emphysema, chronic bronchitis, and smoking status were significantly associated with whether participants reported sleep problems.

**Table 2:** Baseline characteristics of participants with status of reported sleep problem.

Variable	All participants	Have reported sleep problem	Never reported sleep problem	P-value
Age	46.427 ± 0.108	51.544 ± 0.202	44.850 ± 0.125	<0.001
BMI	28.736 ± 0.040	30.225 ± 0.091	28.273 ± 0.043	<0.001
Gender				<0.001
Male	48.751 (0.278)	40.799 (0.273)	51.202 (0.278)	
Female	51.249 (0.278)	59.201 (0.273)	48.798 (0.278)	
Race				<0.001
Mexican American	16.052 (0.204)	14.001 (0.193)	17.162 (0.210)	
Other Hispanic	10.827 (0.173)	11.372 (0.177)	10.549 (0.171)	
Non-Hispanic White	40.129 (0.272)	35.505 (0.266)	42.610 (0.275)	
Non-Hispanic Black	21.607 (0.229)	28.295 (0.251)	17.987 (0.214)	
Other Race	11.386 (0.177)	10.828 (0.173)	11.692 (0.179)	
Education				<0.001
Below high school	25.981 (0.257)	23.986 (0.250)	26.647 (0.259)	
High school/GED	22.619 (0.245)	23.068 (0.247)	22.469 (0.244)	
College or above	51.400 (0.293)	52.945 (0.292)	50.883 (0.293)	
Poverty Level				0.034
<1	23.862 (0.249)	24.943 (0.253)	23.521 (0.248)	
1-3	41.821 (0.288)	41.638 (0.288)	41.879 (0.288)	
>=3	34.317 (0.277)	33.419 (0.276)	34.600 (0.278)	
Marital status				<0.001
Married/living with partner	62.928 (0.271)	57.386 (0.277)	64.646 (0.268)	
Never married	14.284 (0.196)	13.788 (0.193)	14.438 (0.197)	
Widowed/divorced/separated	22.788 (0.235)	28.826 (0.254)	20.915 (0.228)	
Smoking				<0.001
Non-Smoker	23.308 (0.245)	27.727 (0.260)	21.850 (0.240)	
Former Smoker	56.342 (0.288)	47.301 (0.290)	59.324 (0.285)	
Light Smoker	5.097 (0.128)	4.924 (0.125)	5.154 (0.128)	
Moderate Smoker	3.684 (0.109)	3.920 (0.113)	3.606 (0.108)	
Heavy Smoker	11.570 (0.186)	16.129 (0.213)	10.066 (0.175)	
Exercise load				<0.001
Lack of Exercise	47.599 (0.324)	46.293 (0.323)	52.167 (0.324)	
Adequate Exercise	23.274 (0.274)	23.301 (0.274)	23.179 (0.274)	
Highly Active Exercise	29.127 (0.295)	30.406 (0.298)	24.655 (0.280)	
Diatery energy intake				<0.001
Low Energy Intake	42.184 (0.290)	44.190 (0.292)	41.547 (0.289)	
Adequate Energy Intake	36.847 (0.283)	35.561 (0.281)	37.255 (0.284)	
Excessive Energy Intake	20.970 (0.239)	20.249 (0.236)	21.198 (0.240)	
Asthma				<0.001
Yes	14.801 (0.197)	22.717 (0.233)	12.362 (0.183)	
No	85.199 (0.197)	77.283 (0.233)	87.638 (0.183)	
Diabetes				<0.001
Yes	11.898 (0.182)	18.412 (0.215)	9.921 (0.166)	
No	88.102 (0.182)	81.588 (0.215)	90.079 (0.166)	
Heart Failure				<0.001
Yes	3.339 (0.105)	6.389 (0.136)	2.322 (0.084)	
No	96.661 (0.105)	93.611 (0.136)	97.678 (0.084)	
Emphysema				<0.001
Yes	2.127 (0.085)	6.389 (0.136)	2.322 (0.084)	
No	97.873 (0.085)	93.610 (0.136)	97.678 (0.084)	
Chronic Bronchitis				<0.001
Yes	5.513 (0.134)	11.079 (0.174)	3.657 (0.104)	
No	94.487 (0.134)	88.921 (0.174)	96.343 (0.104)	

#### 3.1.3 Baseline data for snoring

**Table 3** presents the weighted baseline characteristics of participants who never snore, rarely snore, occasionally snore, and frequently snore. Among the analyzed sample subjects, age, BMI, gender, race, education level, poverty level, marital status, asthma, diabetes, heart failure, chronic bronchitis and smoking status were significantly associated with the distribution of snoring frequency.

**Table 3:** Baseline characteristics of participants by snoring frequency.

Variable	Never	Rarely	Occasionally	Frequently	P value
Age	41.256 ± 0.604	43.417 ± 0.435	46.614 ± 0.733	46.574 ± 0.412	<0.001
BMI	26.435 ± 0.191	27.555 ± 0.298	29.385 ± 0.207	31.552 ± 0.200	<0.001
Gender					<0.001
Male	36.921 (1.275)	43.459 (1.241)	48.389 (1.315)	56.786 (1.320)	
Female	63.079 (1.275)	56.541 (1.241)	51.611 (1.315)	43.214 (1.320)	
Race					<0.001
Mexican American	8.651 (1.463)	9.186 (1.672)	9.598 (1.500)	9.990 (1.643)	
Other Hispanic	13.502 (1.746)	11.621 (1.640)	12.675 (1.567)	12.266 (1.677)	
Non-Hispanic White	62.147 (3.091)	65.124 (2.599)	63.222 (2.887)	61.980 (3.788)	
Non-Hispanic Black	6.472 (0.934)	4.407 (0.593)	5.509 (0.974)	7.631 (1.374)	
Other Race	9.227 (1.426)	9.662 (1.133)	8.996 (1.364)	8.133 (1.091)	
Education					<0.001
Below high school	17.068 (1.298)	14.377 (1.467)	16.540 (1.429)	20.344 (1.433)	
High school/GED	23.028 (1.145)	18.587 (1.298)	22.226 (1.588)	25.664 (1.611)	
College or above	59.904 (1.680)	67.036 (2.320)	61.234 (2.310)	53.993 (2.287)	
Poverty Level					<0.001
<1	18.385 (1.327)	14.004 (1.220)	13.143 (1.361)	15.528 (1.440)	
1-3	38.406 (1.645)	32.911 (1.927)	36.358 (2.231)	38.235 (1.795)	
>=3	43.209 (2.144)	53.085 (2.566)	50.500 (2.520)	46.238 (2.379)	
Marital status					<0.001
Married/living with partner	63.521 (1.813)	74.472 (1.477)	73.057 (1.676)	71.753 (1.476)	
Never married	17.058 (1.156)	11.974 (1.011)	11.187 (1.301)	9.859 (0.984)	
Widowed/divorced/separated	19.421 (1.392)	13.554 (1.180)	15.756 (1.015)	18.389 (1.069)	
Smoking					<0.001
Non-Smoker	61.961 (1.728)	59.514 (1.532)	59.324 (0.285)	47.298 (1.510)	
Former Smoker	20.243 (0.888)	23.013 (1.135)	21.850 (0.240)	27.760 (1.158)	
Light Smoker	4.576 (0.637)	4.512 (0.467)	5.154 (0.128)	4.874 (0.485)	
Moderate Smoker	3.602 (0.338)	2.738 (0.384)	3.606 (0.108)	2.854 (0.319)	
Heavy Smoker	9.619 (1.051)	10.223 (0.928)	10.066 (0.175)	17.213 (1.422)	
Exercise load					0.083
Lack of Exercise	43.553 (1.260)	41.903 (1.798)	41.111 (1.661)	43.730 (1.606)	
Adequate Exercise	25.687 (1.083)	25.560 (1.301)	23.875 (1.604)	21.486 (1.233)	
Highly Active Exercise	30.760 (1.470)	32.537 (1.752)	35.014 (1.691)	34.783 (1.517)	
Diatery energy intake					0.073
Low Energy Intake	40.214 (1.541)	37.775 (1.115)	35.765 (1.602)	36.555 (1.485)	
Adequate Energy Intake	38.492 (1.150)	40.307 (1.072)	41.551 (1.872)	38.545 (1.185)	
Excessive Energy Intake	21.294 (0.890)	21.917 (1.141)	22.685 (1.203)	24.899 (1.252)	
Asthma					0.022
Yes	15.625 (0.763)	15.997 (0.869)	12.642 (0.955)	16.024 (0.681)	
No	84.375 (0.763)	84.003 (0.869)	87.358 (0.955)	83.976 (0.681)	
Diabetes					<0.001
Yes	7.033 (0.634)	7.382 (0.675)	11.031 (1.112)	12.299 (0.992)	
No	92.967 (0.634)	92.618 (0.675)	88.969 (1.112)	87.701 (0.992)	
Heart Failure					0.006
Yes	2.303 (0.313)	1.271 (0.234)	2.739 (0.448)	2.785 (0.375)	
No	97.697 (0.313)	98.729 (0.234)	97.261 (0.448)	97.215 (0.375)	
Emphysema					0.184
Yes	1.721 (0.251)	1.491 (0.339)	1.943 (0.344)	2.335 (0.286)	
No	98.279 (0.251)	98.509 (0.339)	98.057 (0.344)	97.665 (0.286)	
Chronic Bronchitis					0.006
Yes	5.715 (0.753)	4.671 (0.618)	5.554 (0.651)	7.740 (0.813)	
No	94.285 (0.753)	95.329 (0.618)	94.446 (0.651)	92.260 (0.813)	

#### 3.1.4 Baseline data for snort or stop breathing

**Table 4** presents the weighted baseline characteristics of participants who never, rarely, occasionally, and frequently experience snorting or stopping breathing during sleep. Among the analyzed sample subjects, age, BMI, gender, education level, poverty level, marital status, asthma, diabetes, heart failure, emphysema, chronic bronchitis, and smoking status were significantly associated with the distribution of snorting or stopping breathing frequency.

**Table 4:** Baseline characteristics of participants by snorting or stopping breathing frequency.

Variable	Never	Rarely	Occasionally	Frequently	P value
Age	43.636 ± 0.404	47.213 ± 1.004	49.905 ± 0.588	48.859 ± 0.766	<0.001
BMI	27.977 ± 0.152	29.819 ± 0.268	30.177 ± 0.368	33.318 ± 0.370	<0.001
Gender					<0.001
Male	0.461 (0.005)	0.565 (0.021)	0.621 (0.023)	0.582 (0.032)	
Female	0.539 (0.005)	0.435 (0.021)	0.379 (0.023)	0.418 (0.032)	
Race					0.241
Mexican American	8.819 (1.328)	9.867 (1.932)	9.020 (1.287)	6.527 (1.191)	
Other Hispanic	11.577 (1.457)	11.000 (1.886)	10.906 (1.603)	8.723 (1.777)	
Non-Hispanic White	65.695 (2.707)	64.963 (2.933)	66.190 (3.392)	71.893 (3.679)	
Non-Hispanic Black	5.786 (0.859)	6.297 (1.259)	5.627 (1.423)	6.616 (1.256)	
Other Race	8.122 (0.919)	7.874 (1.209)	8.256 (1.542)	6.241 (1.039)	
Education					<0.001
Below high school	16.331 (1.120)	16.942 (1.607)	20.216 (2.045)	18.262 (2.282)	
High school/GED	21.974 (0.922)	23.802 (2.237)	25.591 (2.153)	26.824 (2.567)	
College or above	61.695 (1.752)	59.255 (2.722)	54.193 (3.397)	54.914 (3.061)	
Poverty Level					<0.001
<1	14.411 (0.901)	15.346 (1.767)	15.551 (2.036)	13.846 (2.021)	
1-3	36.617 (1.418)	35.845 (2.371)	38.519 (2.515)	35.416 (2.708)	
>=3	48.972 (1.979)	48.809 (2.430)	45.930 (2.761)	50.737 (3.237)	
Marital status					<0.001
Married/living with partner	70.593 (1.021)	73.943 (1.957)	76.011 (2.247)	69.143 (3.351)	
Never married	12.701 (0.796)	9.117 (1.166)	7.687 (1.195)	7.671 (1.590)	
Widowed/divorced/separated	16.706 (0.800)	16.940 (1.617)	16.302 (1.874)	23.187 (2.869)	
Smoking					<0.001
Non-Smoker	57.827 (1.112)	53.544 (1.585)	48.564 (2.395)	44.215 (3.008)	
Former Smoker	23.148 (0.664)	26.602 (1.897)	23.369 (1.922)	29.979 (2.792)	
Light Smoker	4.605 (0.329)	5.199 (0.719)	6.670 (1.433)	3.261 (0.915)	
Moderate Smoker	2.943 (0.233)	3.827 (0.912)	3.233 (0.750)	2.591 (0.666)	
Heavy Smoker	11.478 (0.798)	10.827 (1.117)	18.165 (2.365)	19.954 (2.749)	
Exercise load					0.700
Lack of Exercise	42.535 (0.010)	42.282 (0.024)	41.584 (0.027)	43.433 (0.033)	
Adequate Exercise	24.694 (0.007)	22.665 (0.023)	21.706 (0.023)	25.425 (0.034)	
Highly Active Exercise	32.771 (0.012)	35.053 (0.024)	36.709 (0.026)	31.142 (0.039)	
Diatery energy intake					0.491
Low Energy Intake	38.504 (0.010)	36.023 (0.021)	38.298 (0.021)	34.127 (0.024)	
Adequate Energy Intake	39.414 (0.008)	40.833 (0.021)	40.699 (0.030)	39.431 (0.026)	
Excessive Energy Intake	22.083 (0.007)	23.144 (0.021)	21.003 (0.023)	26.442 (0.024)	
Asthma					0.033
Yes	14.465 (0.518)	17.506 (1.355)	17.007 (1.776)	18.790 (2.202)	
No	85.535 (0.518)	82.495 (1.355)	82.993 (1.776)	81.210 (2.202)	
Diabetes					<0.001
Yes	7.937 (0.451)	11.219 (1.111)	11.488 (1.320)	21.451 (2.774)	
No	92.063 (0.451)	88.781 (1.111)	88.512 (1.320)	78.549 (2.774)	
Heart Failure					0.001
Yes	1.921 (0.205)	2.638 (0.542)	3.284 (0.968)	5.086 (1.040)	
No	98.079 (0.205)	97.362 (0.542)	96.716 (0.968)	94.914 (1.040)	
Emphysema					0.184
Yes	1.555 (0.208)	2.541 (0.367)	3.526 (1.097)	3.045 (0.752)	
No	98.445 (0.208)	97.459 (0.367)	96.474 (1.097)	96.955 (0.752)	
Chronic Bronchitis					<0.001
Yes	4.941 (0.534)	6.675 (0.795)	9.829 (1.516)	11.468 (1.692)	
No	95.059 (0.534)	93.325 (0.795)	90.171 (1.516)	88.532 (1.692)	

#### 3.1.5 Baseline data for feeling overly sleepy during the day

**Table 5** presents the weighted baseline characteristics of participants who never, rarely, sometimes, often, and almost always feel overly sleepy during the day. Among the analyzed sample subjects, age, BMI, gender, race, education level, poverty level, dietary energy intake, asthma, diabetes, heart failure, emphysema, chronic bronchitis and smoking status were significantly associated with the distribution of feeling overly sleepy frequency.

**Table 5:** Baseline characteristics of participants by feeling overly sleepy during the day.

Variable	Never	Rarely	Sometimes	Often	Almost always	P value
Age	46.457 ± 0.394	45.784 ± 0.685	44.603 ± 0.462	42.840 ± 0.760	43.072 ± 0.841	<0.001
BMI	27.819 ± 0.181	28.344 ± 0.186	28.900 ± 0.188	29.347 ± 0.234	30.320 ± 0.488	<0.001
Gender						<0.001
Male	53.150 (0.925)	49.769 (1.180)	49.931 (1.076)	43.998 (1.733)	39.113 (2.027)	
Female	46.850 (0.925)	50.231 (1.180)	50.069 (1.076)	56.002 (1.733)	60.887 (2.027)	
Race						<0.001
Mexican American	12.600 (1.970)	8.417 (1.283)	7.892 (1.364)	6.631 (1.073)	7.218 (1.380)	
Other Hispanic	13.526 (1.631)	10.466 (1.471)	11.241 (1.716)	10.120 (1.719)	10.882 (1.861)	
Non-Hispanic White	55.920 (3.658)	68.302 (2.616)	68.244 (2.840)	71.843 (2.602)	67.540 (3.474)	
Non-Hispanic Black	8.028 (1.588)	5.564 (0.852)	4.783 (0.801)	4.569 (0.832)	7.010 (0.969)	
Other Race	9.931 (1.394)	7.252 (0.948)	7.840 (0.977)	6.836 (0.878)	7.350 (1.336)	
Education						<0.001
Below high school	24.304 (1.438)	14.115 (1.449)	14.332 (1.247)	13.578 (1.313)	20.056 (1.811)	
High school/GED	24.313 (1.035)	21.698 (1.309)	22.291 (1.363)	22.944 (1.461)	57.935 (2.857)	
College or above	51.383 (1.645)	64.187 (2.201)	63.377 (2.127)	63.478 (1.969)	22.010 (2.328)	
Poverty Level						<0.001
<1	18.042 (1.289)	11.126 (0.842)	13.068 (1.085)	14.469 (1.237)	21.103 (2.162)	
1-3	39.54 (1.797)	33.358 (1.879)	34.973 (1.963)	39.634 (2.19)	40.893 (2.239)	
>=3	42.418 (2.301)	55.516 (2.206)	51.959 (2.552)	45.897 (2.605)	38.004 (2.526)	
Marital status						0.152
Married/living with partner	71.112 (1.346)	72.81 (1.516)	71.221 (1.436)	67.573 (2.035)	68.457 (2.171)	
Never married	10.751 (0.857)	11.32 (1.008)	11.96 (0.905)	13.737 (1.619)	11.558 (1.417)	
Widowed/divorced/separated	18.138 (1.094)	15.87 (1.176)	16.82 (1.225)	18.69 (1.499)	19.986 (1.500)	
Smoking						<0.001
Non-Smoker	57.924 (1.768)	58.039 (1.701)	56.847 (1.2)	52.322 (1.517)	43.319 (2.237)	
Former Smoker	24.317 (1.116)	25.216 (1.433)	22.957 (1.043)	24.179 (1.137)	25.761 (2.330)	
Light Smoker	4.506 (0.502)	3.899 (0.548)	4.512 (0.657)	5.358 (0.589)	6.614 (1.425)	
Moderate Smoker	2.865 (0.323)	2.366 (0.467)	3.459 (0.406)	3.603 (0.484)	2.443 (0.529)	
Heavy Smoker	10.388 (0.983)	10.48 (0.937)	12.224 (1.035)	14.539 (1.223)	21.864 (2.395)	
Exercise load						0.288
Lack of Exercise	43.798 (1.363)	41.185 (1.790)	43.775 (1.388)	43.100 (1.536)	40.087 (2.746)	
Adequate Exercise	24.638 (1.242)	25.352 (1.569)	24.569 (0.946)	21.432 (1.465)	25.467 (1.713)	
Highly Active Exercise	31.565 (1.372)	33.463 (1.523)	31.656 (1.396)	35.468 (1.676)	34.446 (2.819)	
Diatery energy intake						<0.001
Low Energy Intake	44.530 (1.289)	38.491 (1.171)	35.916 (1.182)	34.025 (1.981)	39.060 (2.690)	
Adequate Energy Intake	35.817 (1.070)	40.533 (1.179)	41.468 (1.067)	40.612 (1.656)	34.503 (3.169)	
Excessive Energy Intake	19.653 (1.195)	20.976 (1.303)	22.617 (0.713)	25.363 (1.695)	26.437 (2.190)	
Asthma						<0.001
Yes	11.013 (0.851)	13.143 (1.015)	15.744 (0.816)	20.899 (1.174)	22.258 (1.984)	
No	88.987 (0.851)	86.857 (1.015)	84.256 (0.816)	79.101 (1.174)	77.742 (1.984)	
Diabetes						<0.001
Yes	9.054 (0.743)	8.270 (0.656)	9.137 (0.624)	9.598 (1.060)	15.240 (2.065)	
No	90.946 (0.743)	91.730 (0.656)	90.863 (0.624)	90.402 (1.060)	84.760 (2.065)	
Heart Failure						<0.001
Yes	2.054 (0.313)	1.788 (0.216)	2.248 (0.251)	2.466 (0.264)	4.867 (1.075)	
No	97.946 (0.313)	98.212 (0.216)	97.752 (0.251)	97.534 (0.264)	95.133 (1.075)	
Emphysema						<0.001
Yes	1.414 (0.261)	1.144 (0.292)	1.783 (0.265)	3.135 (0.458)	4.396	
No	98.586 (0.261)	98.856 (0.292)	98.217 (0.265)	96.865 (0.458)	95.604 (0.836)	
Chronic Bronchitis						<0.001
Yes	3.398 (0.566)	4.568 (0.592)	6.146 (0.854)	7.687 (0.946)	13.378 (1.977)	
No	96.602 (0.566)	95.432 (0.592)	93.854 (0.854)	92.313 (0.946)	86.622 (1.977)	

### 3.2 The association between smoking categories and sleep disorder outcomes

**Table 6** presents the weighted associations between smoking status and various sleep disorders outcomes, including insufficient sleep duration, reported sleep problems, snoring, snorting or stopping breathing during sleep, and feeling overly sleepy, after adjusting for confounders. Crude models and models adjusted for age, gender, and race are provided in the **Supplementary Data** (**Supplementary Tables S2–6**).

**Table 6:** Adjusted associations between smoking levels and insufficient sleep duration, reported sleep problems, snoring, snorting or stop breathing, and feeling overly sleepy during the day.

Smoking category	Insufficient Sleep Duration		Reported Sleep problems		Snoring		Snort or Stop Breathing		Feeling Overly Sleepy	
	OR (95% CI)	P-value	OR (95% CI)	P-value	OR (95% CI)	P-value	OR (95% CI)	P-value	OR (95% CI)	P-value
Non Smoker	Reference		Reference		Reference		Reference		Reference	
Former Smoker	1.032 (0.938, 1.136)	0.508	1.435 (1.310, 1.573)	<0.001	1.177 (0.818, 1.716)	0.204	1.039 (0.707, 1.525)	0.774	1.023 (0.767, 1.363)	0.769
Light Smoker	1.057 (0.901, 1.362)	0.49	1.364 (1.113, 1.672)	0.004	1.320 (0.738, 2.361)	0.176	1.456 (0.655, 3.239)	0.232	1.259 (0.683, 2.321)	0.247
Moderate Smoker	1.272 (1.047, 1.546)	0.017	1.749 (1.398, 2.186)	<0.001	1.182 (0.585, 2.387)	0.414	1.689 (0.794, 3.591)	0.114	1.462 (0.740, 2.887)	0.139
Heavy Smoker	1.732 (1.528, 1.963)	<0.001	1.990 (1.766, 2.243)	<0.001	1.908 (1.164, 3.128)	0.03	1.863 (1.183, 2.936)	0.022	1.257 (0.872, 1.810)	0.115

Considered covariates including age, gender, race, BMI, education level, marital status, poverty level, physical activity level, dietary energy intake, and medication status including asthma, diabetes, heart failure, emphysema, and chronic bronchitis.

Compared to non-smokers, former smokers had significantly higher odds of reporting sleep problems (OR: 1.435, 95% CI: 1.310–1.573, P<0.001). Light smokers exhibited significantly higher odds of reporting sleep problems (OR: 1.364, 95% CI: 1.113–1.672, P=0.004) compared to non-smokers. Moderate smokers had significantly higher odds of insufficient sleep duration (OR: 1.272, 95% CI: 1.047–1.546, P=0.017) and reported sleep problems (OR: 1.749, 95% CI: 1.398–2.186, P<0.001) compared to non-smokers. Heavy smokers consistently demonstrated significant associations across multiple sleep outcomes. They had higher odds of insufficient sleep duration (OR: 1.732, 95% CI: 1.528–1.963, P<0.001), reported sleep problems (OR: 1.990, 95% CI: 1.766–2.243, P<0.001), snoring (OR: 1.908, 95% CI: 1.164–3.128, P=0.03), and snorting or stopping breathing during sleep (OR: 1.863, 95% CI: 1.183–2.936, P=0.022) compared to non-smokers. However, no significant association was found for feeling overly sleepy.

### 3.3 Smoking trend and sleep disorder outcomes

**Table 7** presents the associations between smoking trend and various sleep-related outcomes. Compared to non-smokers, smokers had a higher odds of reporting insufficient sleep duration (OR: 1.112, 95% CI: 1.088–1.136, P<0.001). Similarly, smokers were more likely to report sleep problems (OR: 1.187, 95% CI: 1.159–1.215, P<0.001) and snoring (OR: 1.154, 95% CI: 1.114–1.195, P<0.001) than non-smokers. The odds of experiencing snorting or stopping breathing during sleep were also higher among smokers (OR: 1.172, 95% CI: 1.116–1.231, P<0.001). Additionally, smokers had a higher likelihood of feeling overly sleepy during the day compared to non-smokers (OR: 1.079, 95% CI: 1.043–1.116, P<0.001).

**Table 7:** Associations between smoking trend and sleep-related outcomes.

Outcome Categories	Smoking Trend		
	Odd Raio (OR)	95% CI	P-value
Insufficient sleep Duration	1.112	(1.088, 1.136)	<0.001
Reported Sleep problems	1.187	(1.159, 1.215)	<0.001
Snoring	1.154	(1.114, 1.195)	<0.001
Snort or Stop Breathing	1.172	(1.116, 1.231)	<0.001
Feeling Overly Sleepy	1.079	(1.043, 1.116)	<0.001

### 3.4 Dose-response curve for sleeping disorder outcomes

**Figure 2:**
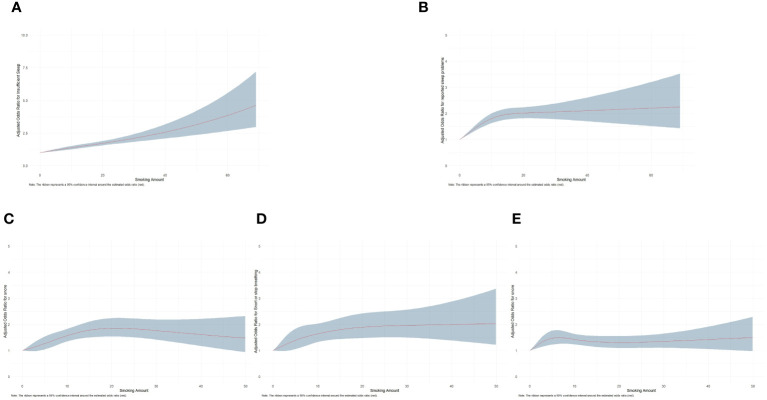
Dose-response relationship between smoking amount and **(A)** sleep insufficiency, **(B)** reported sleep problems, **(C)** snoring at a high frequency, **(D)** snorting or stopping breathing at a high frequency, and **(E)** feeling overly sleepy during the day at a high frequency. The results were obtained using restricted cubic splines. The red lines indicate the estimated odds ratios, and the blue regions indicate the 95% confidence intervals.

**Figure 2** presents dose-response curves showing an increase in the adjusted odds ratios for various sleep outcomes, including insufficient sleep, reported sleep problems, snoring, snorting or stopping breathing, and feeling overly sleepy during the day, as the amount of smoking rises. The dose-response curves reveal that when the daily smoking amount is less than approximate 30 cigarettes, the risk of these sleep problems increases with the rise in smoking quantity, suggesting a dose-dependent adverse effect of smoking on sleep. However, when the daily smoking amount exceeds approximate 30 cigarettes, the curves exhibit a flattening or declining trend, accompanied by widening confidence intervals. This might be attributed to the relatively smaller sample size within the high smoking quantity range, leading to increased uncertainty in the estimates. The expanding confidence intervals suggest greater variability in the odds ratio estimates at higher smoking levels, but the overall patterns remain consistent.

## 4 Discussion

Several previous research have investigated the relationship between sleep disorder and smoking, and smoking have been shown to lead to several sleep disorder outcomes, including insomnia, sleep disturbances and poor sleep quality (11, 12, 19–21). This large, nationally representative cross-sectional study found significant associations between smoking and a variety of sleep outcomes, including insufficient sleep duration, sleep problems, snoring, snorting or stopping breathing during sleep, and feeling overly sleepy during the day. Dose-response analyses revealed that a higher frequency of smoking was associated with a greater probability of poor sleep outcomes, which is consistent with previous findings. However, compared to previous research, our study provides more conclusive evidence due to the large sample size. We also considered more sleep outcomes, which provides a more complete picture of the impact of smoking on sleep health. We also conducted dose-response analyses to quantify the relationship between smoking amount and the probability of poor sleep outcomes. These findings underscore the importance of addressing smoking as a modifiable risk factor for sleep disorders, which can have significant implications for public health.

Several potential mechanisms may underlie the link between smoking and sleep disturbances. Nicotine, the main stimulant in cigarettes, can increase arousal, leading to withdrawal symptoms during the nighttime (22, 23). Previous research has found that smoking has various negative impacts on the central nervous system, exacerbating neurodegenerative diseases, insomnia, and cerebrovascular diseases (24). Smoking also affects circadian rhythms, especially since smokers typically have lower melatonin levels, this effect may further disrupt their sleep-wake cycle (25). Nicotine also interferes with acetylcholine pathway, which further disrupt the sleep-wake cycles (26). Smoking also activates inflammatory pathways in the body, leading to a chronic state of systemic inflammation associated with sleep disorders (27, 28). Furthermore, smoking has been associated with various mental health problems such as anxiety and depression, which are two major risk factors for sleep disorders (29). Chronic diseases caused by smoking, such as chronic obstructive pulmonary disease (COPD) and cardiovascular disease, may also affect sleep quality through multiple mechanisms (30, 31). Lastly, the act of smoking itself may affect sleep hygiene and sleep habits, as many smokers smoke before bedtime or upon awakening during the night, which can delay sleep onset or disrupt sleep (32).

The dose-response relationship indicates that while complete cessation of smoking is the ideal goal, reducing the amount of smoking can still positively affect sleep quality and reduce the risk of sleep disorders for those who are unable to quit entirely. Previous studies have suggested that cessation rather than reduction of smoking can improve health outcomes such as cardiovascular status (33, 34). However, our research suggests that, at least for sleep disorders, reducing the amount of smoking may also improve the consequences, providing a rationale for developing more flexible and realistic public health strategies. These findings can inform the development of targeted interventions and smoking cessation programs that emphasize the benefits of reducing smoking, even if complete abstinence is not achievable. Such strategies may include gradual reduction approaches and nicotine replacement therapies to help smokers improve their sleep quality while working towards the ultimate goal of quitting (35, 36).

Our study has several strengths, including the use of a nationally representative sample, examination of multiple sleep outcomes, and exploration of dose-response relationships. Our findings have important implications for public health and clinical practice, and addressing smoking could be a key strategy for improving sleep health in populations. However, this research also has some limitations. One of the limitations of this study is its cross-sectional design, which precludes the establishment of causal relationships between smoking and sleep outcomes. Additionally, the use of self-reported smoking status may introduce potential biases, such as recall bias or social desirability bias, leading to underreporting or overreporting of smoking, which could affect the accuracy of the results (37, 38). Finally, although we adjusted for several important confounders, the possibility of residual confounding by unmeasured factors cannot be entirely ruled out. Another limitation is that the sample volume of subjects who have a high daily cigarette consumption is low, leading to an unexplored relationship between smoking and sleep outcomes in the dose-response curves when the smoking level is high. In the future, research could focus on subjects with high smoking levels to explore the underlying relationship further.

In conclusion, this study found significant associations and a dose-response relationship between smoking and various sleep problems. These findings underscore the importance of addressing smoking to improve sleep health and reduce the risk of related health problems. As part of sleep disorder management, healthcare providers should routinely assess smoking status and provide support for quitting or reducing smoking. Public health campaigns should also include information about the negative impact of smoking on sleep and the potential benefits of smoking reduction.

## Data availability statement

Publicly available datasets were analyzed in this study. This data can be found here: https://www.cdc.gov/nchs/nhanes/index.htm.

## Ethics statement

The studies involving humans were approved by National Center for Health Statistics (NCHS) Ethics Review Board (ERB). The studies were conducted in accordance with the local legislation and institutional requirements. Written informed consent for participation was not required from the participants or the participants’ legal guardians/next of kin in accordance with the national legislation and institutional requirements.

## Author contributions

HS: Conceptualization, Data curation, Formal analysis, Funding acquisition, Investigation, Methodology, Project administration, Resources, Software, Supervision, Validation, Visualization, Writing – original draft, Writing – review & editing. SL: Writing – original draft, Writing – review & editing.
