# Supplementary material for: Exploring the relationship between smoking and poor sleep quality: a cross-sectional study using NHANES
Source: Front Psychiatry. 2024 May 28;15:1407741. doi: 10.3389/fpsyt.2024.1407741 (PMC11212010; doi:10.3389/fpsyt.2024.1407741)
Supplement: Supplementary file 1 [file Table_1.docx]

**Supplementary Table S1. The comparison of subjects before and after exclusion for baseline data.**

**Supplementary Table S2. Associations between smoking levels and insufficient sleep duration.**

Crude model: no covariates were considered. Model 1: adjusted for age, gender, and race. Model 2: further adjusted for BMI, education level, marital status, poverty level, exercise load, diatery energy intake, asthma, diabetes, heart failure, emphysema adn chronic bronchitis.

**Supplementary Table S3. Associations between smoking levels and reported sleep problem status.**

Crude model: no covariates were considered. Model 1: adjusted for age, gender, and race. Model 2: further adjusted for BMI, education level, marital status, poverty level, exercise load, diatery energy intake, asthma, diabetes, heart failure, emphysema adn chronic bronchitis.

**Supplementary Table S4. Associations between smoking levels and occasional or frequent snoring**

Crude model: no covariates were considered. Model 1: adjusted for age, gender, and race. Model 2: further adjusted for BMI, education level, marital status, poverty level, exercise load, diatery energy intake, asthma, diabetes, heart failure, emphysema adn chronic bronchitis.

**Supplementary Table S5. Associations between smoking levels and occasional or frequent snorting or stopping breathing.**

Crude model: no covariates were considered. Model 1: adjusted for age, gender, and race. Model 2: further adjusted for BMI, education level, marital status, poverty level, exercise load, diatery energy intake, asthma, diabetes, heart failure, emphysema adn chronic bronchitis.

**Supplementary Table S6. Associations between smoking levels and sometimes, often, or almost always feeling overly sleepy during the day.**

Crude model: no covariates were considered. Model 1: adjusted for age, gender, and race. Model 2: further adjusted for BMI, education level, marital status, poverty level, exercise load, diatery energy intake, asthma, diabetes, heart failure, emphysema adn chronic bronchitis.
